# GDF‐15 is a better complimentary marker for risk stratification of arrhythmic death in non‐ischaemic, dilated cardiomyopathy than soluble ST2

**DOI:** 10.1111/jcmm.13540

**Published:** 2018-02-04

**Authors:** Stefan Stojkovic, Alexandra Kaider, Lorenz Koller, Mira Brekalo, Johann Wojta, Andre Diedrich, Svitlana Demyanets, Thomas Pezawas

**Affiliations:** ^1^ Department of Internal Medicine II Division of Cardiology Medical University of Vienna Vienna Austria; ^2^ Ludwig Boltzmann Cluster for Cardiovascular Research Vienna Austria; ^3^ Center for Medical Statistics, Informatics, and Intelligent Systems – Section for Clinical Biometrics Medical University of Vienna Vienna Austria; ^4^ Core Facilities Medical University of Vienna Vienna Austria; ^5^ Department of Medicine Division of Clinical Pharmacology Autonomic Dysfunction Center Vanderbilt University Medical Center Nashville TN USA; ^6^ Department of Laboratory Medicine Medical University of Vienna Vienna Austria

**Keywords:** arrhythmic death, GDF‐15, heart failure, sST2, sudden death

## Abstract

Growth differentiation factor (GDF)‐15 and soluble ST2 (sST2) are established prognostic markers in acute and chronic heart failure. Assessment of these biomarkers might improve arrhythmic risk stratification of patients with non‐ischaemic, dilated cardiomyopathy (DCM) based on left ventricular ejection fraction (LVEF). We studied the prognostic value of GDF‐15 and sST2 for prediction of arrhythmic death (AD) and all‐cause mortality in patients with DCM. We prospectively enrolled 52 patients with DCM and LVEF ≤ 50%. Primary end‐points were time to AD or resuscitated cardiac arrest (RCA), and secondary end‐point was all‐cause mortality. The median follow‐up time was 7 years. A cardiac death was observed in 20 patients, where 10 patients had an AD and 2 patients had a RCA. One patient died a non‐cardiac death. GDF‐15, but not sST2, was associated with increased risk of the AD/RCA with a hazard ratio (HR) of 2.1 (95% CI = 1.1‐4.3; *P* = .031). GDF‐15 remained an independent predictor of AD/RCA after adjustment for LVEF with adjusted HR of 2.2 (95% CI = 1.1‐4.5; *P* = .028). Both GDF‐15 and sST2 were independent predictors of all‐cause mortality (adjusted HR = 2.4; 95% CI = 1.4‐4.2; *P* = .003 vs HR = 1.6; 95% CI = 1.05‐2.7; *P* = .030). In a model including GDF‐15, sST2, LVEF and NYHA functional class, only GDF‐15 was significantly associated with the secondary end‐point (adjusted HR = 2.2; 95% CI = 1.05‐5.2; *P* = .038). GDF‐15 is superior to sST2 in prediction of fatal arrhythmic events and all‐cause mortality in DCM. Assessment of GDF‐15 could provide additional information on top of LVEF and help identifying patients at risk of arrhythmic death.

## 1 INTRODUCTION

Non‐ischaemic, dilated cardiomyopathy (DCM), characterized by left ventricular (LV) dilation and reduced systolic function without relevant coronary artery disease, represents an underlying cause for approximately one‐third of heart failure patients.^1^, ^2^ Progressive heart failure and ventricular tachyarrhythmia (VT) are the most common cause of sudden death in these patients.^3^ Current guidelines for primary prevention with implantable cardioverter defibrillators (ICD) are based on the degree of LV ejection fraction (LVEF ≤ 30%‐40%) reduction.^3^, ^4^ However, patients with ischaemic heart disease seem to have more benefit from prophylactic ICD implantation.^5^, ^6^, ^7^ Furthermore, the recently published DANISH trial has demonstrated no mortality benefit from prophylactic ICD implantation in non‐ischaemic DCM.^8^ Although LVEF is currently the best marker for risk stratification, it lacks specificity and many DCM patients with ICDs never receive appropriate therapies.^9^, ^10^ It is well known that VT also occurs in patients with borderline or mildly reduced LVEF.^3^ Non‐invasive testing and LVEF could not reliably identify patients with DCM at risk of fatal VTs.^11^ Therefore, the identification of additional markers for arrhythmia risk stratification of patients with DCM is essential.

Growth differentiation factor (GDF)‐15 and soluble ST2 (sST2) are well‐established prognostic markers for mortality in acute and chronic heart failure^12^, ^13^, ^14^, ^15^, ^16^, ^17^ as well as in acute myocardial infarction.^18^ Inflammation and myocardial fibrosis, with subsequent ventricular remodelling and impairment of systolic function, are important pathophysiological mechanisms for VTs in patients with DCM.^19^, ^20^ Both GDF‐15 and sST2 show strong correlations with myocardial stress and fibrosis,^21^, ^22^, ^23^ and have been associated with sudden cardiac death in DCM.^24^, ^25^

The aim of the study was a head‐to‐head comparison of GDF‐15 and sST2 for prediction of arrhythmic death (AD) and all‐cause mortality in patients with non‐ischaemic DCM.

## 2 MATERIALS AND METHODS

### 2.1 Study participants

This was a prospective, longitudinal cohort study. A total of 52 consecutive patients with non‐ischaemic DCM were included in the study in 2002 and 2003 at the Medical University of Vienna, Austria. Patients were eligible to participate if they were aged ≥18 years, had a LVEF of ≤50%, had recently undergone coronary angiography with ventriculography as standard use of care independently from study participation, echocardiography, MRI at the physician's discretion, had no history of sustained ventricular arrhythmia or permanent atrial fibrillation and were not dependent on ventricular pacing. Ambulatory ECG, Holter recordings and exercise tests were performed at baseline in all patients. New York Heart Association (NYHA) classification was documented for each patient at the baseline according to ESC and AHA Heart Failure Guidelines: NYHA Class I: no limitation of physical activity; NYHA Class II: slight limitation of physical activity in which ordinary physical activity leads to fatigue, palpitation, dyspnoea, or anginal pain; the person is comfortable at rest; Class III: marked limitation of physical activity in which less‐than‐ordinary activity results in fatigue, palpitation, dyspnoea, or anginal pain; the person is comfortable at rest; Class IV: inability to carry on any physical activity without discomfort but also symptoms of heart failure or the anginal syndrome even at rest, with increased discomfort if any physical activity is undertaken.^4^, ^26^ Patients were followed up between 2003 and 2013. The study was approved by the local ethics committee, and all participants gave written informed consent.

### 2.2 Biomarker measurements

Venous blood samples were obtained from each patient at study admission. EDTA blood samples were immediately centrifuged at 1500 g for 15 minutes, plasma was aliquoted and stored at −80^°^C for further use. The samples did not undergo any freeze‐thaw cycles before the performance of the assays. Plasma concentration of GDF‐15 and sST2 was assessed by specific commercially available enzyme‐linked immunosorbent assays (ELISA). Circulating levels of human GDF‐15 were determined by GDF‐15 Quantikine^®^ ELISA Kit (R&D Systems, Minneapolis, MN, USA).^27^ GDF‐15 Quantikine^®^ ELISA measures natural and recombinant human GDF‐15 and has an intra‐assay variability of 1.8%‐2.8% and an inter‐assay variability of 4.7%‐6.0%. Sensitivity of GDF‐15 Quantikine^®^ ELISA is 4.39 pg/mL and a measurement range is 23.4‐1500 pg/mL. The average recovery of human GDF‐15 in EDTA plasma samples is 97%.

sST2 was quantified using human ST2/IL‐1 R4 DuoSet^®^ ELISA Kit (R&D Systems).^28^, ^29^ ST2/IL‐33R ELISA Kit has an intra‐assay variability of 4.4%‐5.6% and an inter‐assay variability of 5.4%‐7.1%. This ELISA has a sensitivity of 13.5 pg/mL, and it is specific for natural and recombinant human ST2 as well as free ST2 and IL‐33 complexed ST2. Assay range of Human ST2/IL‐33R ELISA is 31.3‐2000 pg/mL.

N‐terminal pro‐brain natriuretic peptide (NT‐proBNP) was assessed using an Elecsys immunoassay on a cobas 8000 system (Roche Diagnostics, Mannheim, Germany). Uric acid measurements were taken using standard laboratory technique (URICASE‐PAP‐Method).

### 2.3 Echocardiography

Echocardiographic examinations following a standard protocol^30^ were conducted using a Vivid ultrasound machine (GE Healthcare, Milwaukee, WI, USA) or an ACUSON Sequia (Acuson, Mountain View, CA, USA). Following the practice at our institution, cardioversion was attempted in all patients. This time window, when the patient was in sinus rhythm, was used for echocardiographic examinations. Interventricular septal thickness was obtained at end‐diastole from two‐dimensional directed M‐mode in the parasternal long axis, according to American Society of Echocardiography guidelines.^31^, ^32^ Two‐dimensional apical 4‐ and 2‐chamber views were used to calculate LVEF using the biplane Simpson's method. An LVEF <55% was considered abnormal. The severity of mitral regurgitation was evaluated semiquantitatively from the area of the regurgitant jet by colour Doppler. LA anteroposterior diameter in parasternal long‐axis view and LA major axis in apical 4‐chamber view were used to calculate LA diameters.

### 2.4 End‐points

The primary end‐point was time to AD or resuscitated cardiac arrest (RCA). All‐cause mortality was the secondary end‐point. Deaths were categorized using an adapted form of the Hinkle classification.^33^ Appropriate ICD therapy without VT acceleration that failed to save the patient's life at the time of arrhythmias was classified as AD.^34^ An RCA was ventricular fibrillation or VT > 240 beats per minute leading to syncope before ICD therapy, and multiple slower VT episodes (electrical storm) leading to syncope and ICD discharge without ICD therapy‐related acceleration. All other ICD therapies because of VT < 240 beats per minute were not taken as surrogate for AD. Study end‐point data were collected through pre‐planned ambulatory visits. In case of non‐appearance study, end‐point data were collected through treating physicians or patients′ relatives, and in case of death through abduction, which is mandatory in Austria.

### 2.5 Statistical analysis

Categorical variables are summarized as counts and percentages and are compared by the χ^2^‐test or by Fisher's exact test as appropriate. Continuous variables are expressed as median and interquartile range (IQR) and compared by the *t*‐test (after log‐transformation of the data) or by the Mann‐Whitney *U*‐test in case of non‐normal distributions. The Bonferroni‐Holm correction was applied to correct for the number of multiple comparisons. Estimating the survival probabilities with respect to the primary end‐point AD/RCA, the competing risk approach was used, considering deaths of other causes as competing events. The Gray's test was performed to test for statistically significant differences. Kaplan‐Meier analysis (log‐rank test) was applied to describe and to test for differences with respect to the secondary end‐point all‐cause mortality. Cox proportional hazard regression analysis was performed to assess the effect of GDF‐15 and sST2 on survival. The Firth correction was used in all regression models to reduce bias in estimates due to the rather small number of events.^35^ Hazard ratios (HR) are given as HR per increase of 1 standard deviation (HR per 1‐SD). Due to their skewed distributions, log2‐transformed values of GDF‐15 and sST2 were used within all regression models. Harrell's C‐statistic was applied to evaluate incremental predictive power of GDF‐15 and sST2 in addition to established clinical risk factors.^36^ Two‐sided *P*‐values of ≤.05 indicated statistical significance. SPSS 20.0 (IBM Corporation, Armonk, NY, USA), SAS version 9.4 (SAS Institute Inc., Cary, NC, USA), and STATA version 12 (StataCorp LLC, College Station, TX, USA) were used for all statistical analyses.

## 3 RESULTS

### 3.1 Study population and clinical outcome

Table 1 depicts baseline clinical characteristics of the study population stratified by the primary and secondary end‐points. A total of 52 patients with LVEF of ≤50% were included in the study. The median age of the study cohort was 57.2 years (IQR 51‐64 years) with 76.9% males. The median LVEF was 32% (IQR 28%‐36%). During a median follow‐up time of 7.03 years, 21 deaths were observed. No patients were lost during follow‐up. Cardiac death was observed in 20 patients, and 1 patient died a non‐cardiac death. Of 20 cardiac deaths, 10 patients died an arrhythmic death and 10 patients died due to pump failure. Nine patients were implanted with a prophylactic ICD, and 10 patients were implanted with a CRT at study entry. The ICD therapy was unable to stop an electrical storm in 4 patients, and those patients died an AD. Reasons for not implanting an ICD during later follow‐up in a patient with LVEF ≤ 30% were patients' refusal or the treatment policy of the attending physician. In addition, 2 ICD patients experienced an RCA. Overall, our primary end‐point was observed in 12 patients (10 AD and 2 RCA). Half of the patients with AD/RCA had a LVEF > 30%, and those patients were equally distributed in subgroups LVEF 31%‐40% and 41%‐50% (Table 1). Two‐thirds of patients who died during the follow‐up (secondary end‐point) had LVEF ≤ 30% and just over 50% had NYHA III functional class. None of the patients with NYHA I functional class died or had an RCA during the follow‐up (Table 1).

**Table 1:** Baseline clinical characteristics of the study population

	Overall (n = 52)	AD/RCA (n = 12)	Dead all‐cause (n = 11)	No event (n = 29)	*P*‐value
Demographics					
Age (years)	57.2 (51‐64)	59 (54.8‐65)	57.2 (48.9‐65.3)	54.8 (50.1‐61.7)	.337
Male sex	40 (76.9)	8 (66.7)	9 (81.8)	23 (79.3)	.621
BMI	28 (25.4‐30.8)	26.8 (24.6‐30.1)	26.2 (21.7‐30.3)	28.4 (26.1‐31.9)	.254
Smoking (Pack‐years)	20 (0‐58)	20 (0‐53.7)	35 (0‐50)	20 (0‐60)	.885
Alcoholic	9 (17.3)	2 (16.7)	3 (27.3)	4 (13.8)	.601
NYHA Class					
I	8 (15.4)	0 (0)	0 (0)	8 (27.6)	<.001
II	29 (55.8)	10 (83.3)	2 (18.2)	17 (58.6)	
III	15 (28.8)	2 (16.7)	9 (81.8)	4 (13.8)	
LVEF					
41%‐50%	8 (15.4)	3 (25)	0 (0)	5 (17.2)	.032
31%‐40%	23 (44.2)	3 (25)	3 (27.3)	17 (58.6)	
≤30%	21 (40.4)	6 (50)	8 (72.7)	7 (24.1)	
Medical history					
Hypertension	44 (84.6)	11 (91.7)	10 (90.9)	23 (79.3)	.492
Hypercholesterolaemia	27 (51.9)	5 (41.7)	6 (54.5)	16 (55.2)	.719
Diabetes mellitus	13 (25)	4 (33.3)	3 (27.3)	6 (20.7)	.683
Stroke	5 (9.6)	1 (8.3)	1 (9.1)	3 (10.3)	.978
Renal disease	15 (28.8)	3 (25)	9 (81.9)	3 (10.3)	<.001
Medication					
Beta blockers	46 (88.5)	10 (83.3)	9 (81.8)	27 (93.1)	.497
Amiodarone	8 (15.4)	4 (33.3)	1 (9.1)	3 (10.3)	.144
Sotalol	1 (1.9)	1 (8.3)	0 (0)	0 (0)	.183
ACE‐Inhibitors	44 (84.6)	12 (100)	9 (81.8)	23 (79.3)	.238
ARB‐blockers	18 (34.6)	3 (25)	3 (27.3)	12 (41.4)	.512
Digoxin	14 (26.9)	5 (41.7)	4 (36.4)	5 (17.2)	.201
Diuretics	32 (61.5)	6 (50)	9 (81.8)	17 (58.6)	.260
Device					
ICD	9 (17.3)	6 (50)	2 (18.2)	1 (3.4)	.002
CRT	10 (19.2)	1 (8.3)	2 (18.2)	7 (24.1)	.503
Biomarker					
GDF‐15	884 (575‐2139)	1152 (713‐2139)	2570 (1084‐4819)	740.7 (453.5‐983.4)	.002
sST2	19 (11‐28)	24 (10‐29)	36 (19‐105)	12 (10‐22)	.026

BMI, body mass index; LVEF, left ventricular ejection fraction; ACE, angiotensin‐converting enzyme; ARB, angiotensin receptor blockers; ICD, implantable cardioverter defibrillator; CRT, cardiac resynchronization therapy.

Continuous data are shown as median (interquartile range). Dichotomous data are shown as n (%).

### 3.2 Biomarkers and arrhythmic death

**Figure 1:**
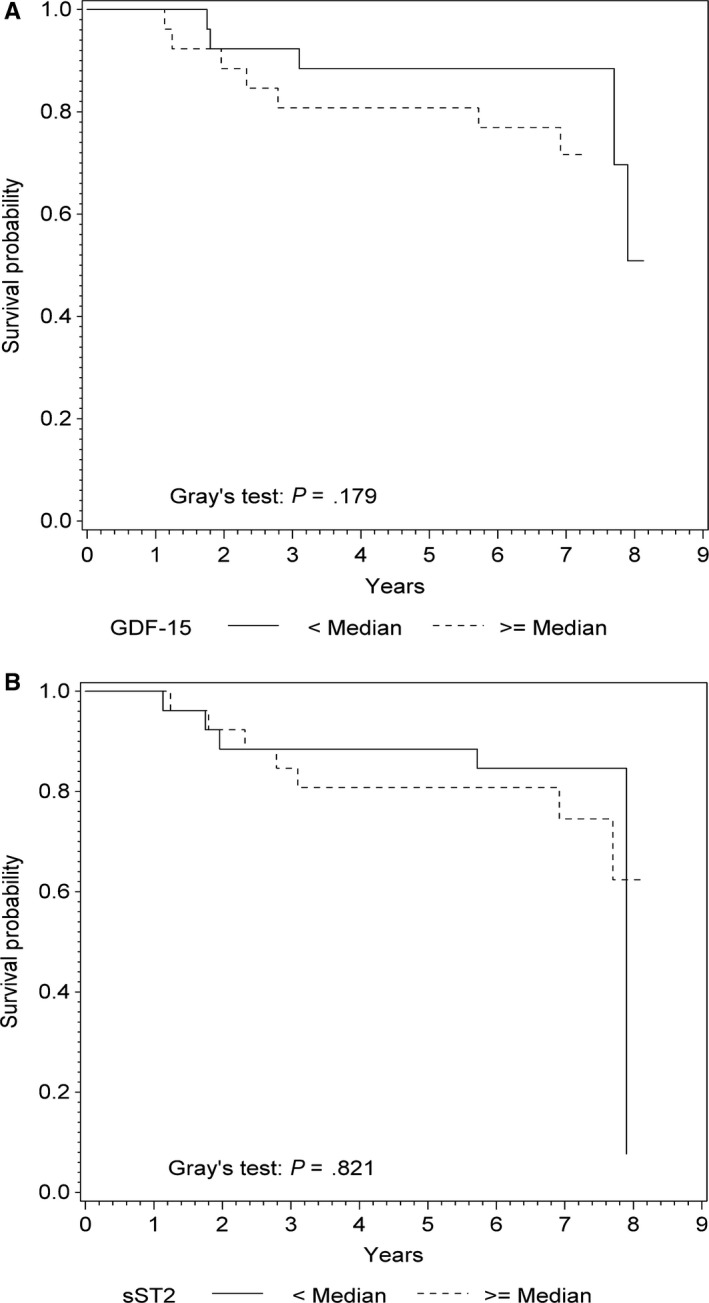
Survival curves for time to arrhythmic death or resuscitated cardiac arrest. A, Time to arrhythmic death or resuscitated cardiac arrest in groups stratified to baseline GDF‐15 above or below the median of 884 pg/mL, accounting for deaths of other causes as competing events. B, Time to arrhythmic death or resuscitated cardiac arrest in groups stratified to sST2 above or below median of 19 ng/mL, accounting for deaths of other causes as competing events

In univariate Cox regression analysis, circulating GDF‐15 was a strong predictor of AD/RCA during the median follow‐up of 7.03 years with a crude HR per increase of 1‐SD of 2.1 (95% CI: 1.1‐4.3; *P* = .031; Table 2). GDF‐15 remained a significant predictor of AD/RCA after adjustment for LVEF (adjusted HR = 2.2; 95% CI: 1.1‐4.5; *P* = .028; Table 2). The area under the curve (AUC, Harrell's C‐statistic) to predict AD/RCA increased from 0.68 (95% CI: 0.55‐0.81) for age, sex and LVEF to 0.76 (95% CI: 0.64‐0.88; *P* = .034; Table 3) when GDF‐15 was added to a model. Figure 1A depicts survival curves for time to AD/RCA, accounting for deaths of other causes as competing events, stratified to baseline GDF‐15 levels above or below the median of 884 pg/mL. There was no association of GDF‐15 above the median and time to AD/RCA (Gray's test: *P* = .179). In contrast to GDF‐15, increased sST2 levels did not predict AD/RCA (HR = 1.5; 95% CI: 0.8‐2.8; *P* = .191; Table 2). As demonstrated in Figure 1B, there was also no association between baseline sST2 levels above the median and time to AD/RCA during the follow‐up (Gray's test: *P* = .821).

**Table 2:** Univariate and multivariable Cox regression analyses for prediction of arrhythmic death/resuscitated cardiac arrest and all‐cause mortality

	Univariate			Multivariable		
	HR per 1‐SD	CI	*P*‐value	HR per 1‐SD	CI	*P*‐value
AD/RCA						
GDF‐15	2.1	1.1‐4.3	.031	2.2^a^	1.1‐4.5	.028
sST2	1.5	0.8‐2.8	.191	1.6^a^	0.8‐3.05	.186
All‐cause mortality						
GDF‐15	3.1	1.9‐5.1	<.001	2.4^b^	1.4‐4.2	.003
				1.8^c^	1.1‐3.0	.025
				2.6^d^	1.6‐4.2	<.001
				2.2^e^	1.05‐5.2	.038
sST2	2.2	1.4‐3.3	<.001	1.6^b^	1.05‐2.7	.030
				1.5^c^	0.9‐2.3	.114
				1.8^d^	1.1‐2.8	.011
				1.04^e^	0.6‐1.9	.907

AD, arrhythmic death; RCA, resuscitated cardiac arrest; HR, hazard ratio; SD, standard deviation; CI, confidence interval.

a Model 1: adjusted for LVEF.

b Model 2: adjusted for LVEF and NYHA functional class.

c Model 3: adjusted for LVEF and NT‐proBNP.

d Model 4: adjusted for LVEF and uric acid.

e Model 5: GDF‐15, sST2, LVEF and NYHA functional class.

**Table 3:** GDF‐15 adds prognostic information on top of clinical risk factors

	AD/RCA			All‐cause mortality		
	C‐index	95% CI	*P*‐value	C‐index	95% CI	*P*‐value
Model 1	0.68	0.55‐0.81		0.72	0.60‐0.84	
Model 1 + GDF‐15	0.76	0.64‐0.88	.034	0.79	0.68‐0.91	.052
Model 1 + sST2	0.69	0.57‐0.82	.409	0.75	0.62‐0.87	.239

Model 1: Area under the curve (Harrell's C‐statistic) for prediction of AD/RCA or all‐cause mortality including age, sex, and left ventricular ejections fraction; AD, arrhythmic death; RCA, resuscitated cardiac arrest; CI, confidence interval.

### 3.3 Biomarkers and all‐cause mortality

**Figure 2:**
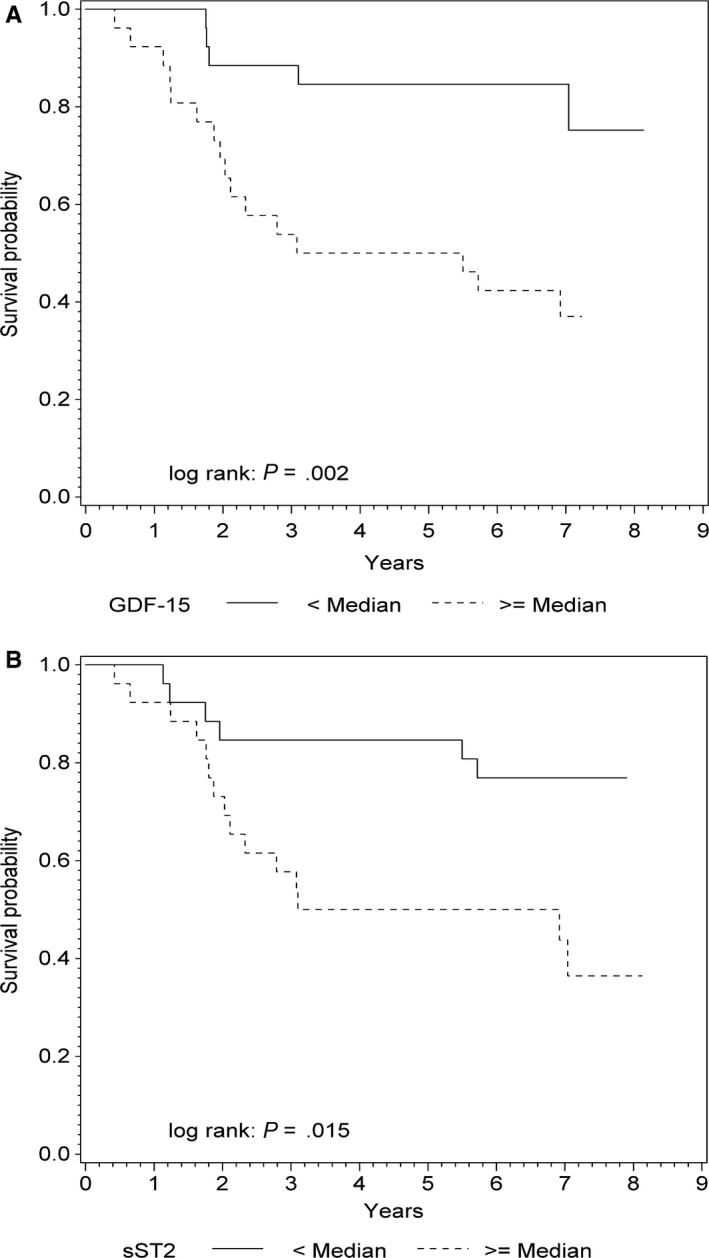
Kaplan‐Meier survival curves for all‐cause mortality. A, Survival in groups according to baseline GDF‐15 above or below median of 884 pg/mL. B, Survival in groups according to baseline sST2 above or below median of 19 ng/mL

Both GDF‐15 and sST2 predicted all‐cause mortality in univariate Cox regression models (HR = 3.1; 95% CI: 1.9‐5.1; *P* < .001 vs HR = 2.2; 95% CI: 1.4‐3.3; *P* < .001; Table 2). Figure 2A,B show corresponding Kaplan‐Maier survival curves of groups stratified according to baseline levels of GDF‐15 and sST2 above or below the median of 884 pg/mL and 19 ng/mL, respectively (log‐rank test: *P* = .002 and *P* = .015).

In a multivariable Cox regression model, including LVEF and NYHA functional class, GDF‐15 was an independent predictor for all‐cause mortality with an adjusted HR of 2.4 (95% CI: 1.4‐4.2; *P* = .003; Table 2). In the same model, sST2 independently predicted all‐cause mortality (adjusted HR = 1.6; 95% CI: 1.05‐2.7; *P* = .030; Table 2). When both GDF‐15 and sST2 were included in a model with LVEF and NYHA functional class, only GDF‐15 remained a significant predictor for all‐cause mortality in patients with non‐ischaemic DCM (adjusted HR = 2.2; 95% CI: 1.05‐5.2; *P* = .038 vs HR = 1.04; 95% CI: 0.6‐1.9; *P* = .907; Table 2). Furthermore, GDF‐15 independently predicted all‐cause mortality after adjustment for NT‐proBNP and uric acid (adjusted HR = 1.8; 95% CI: 1.1‐3.0; *P* = .025 and adjusted HR = 2.6; 95% CI: 1.6‐4.2; *P* < .001, respectively; Table 2). In contrast, sST2 independently predicted all‐cause mortality after adjustment for uric acid (adjusted HR=1.8; 95% CI: 1.1‐2.8; *P* = .011; Table 2), but not after adjustment for NT‐proBNP (adjusted HR=1.5; 95% CI: 0.9‐2.3; *P* = .114; Table 2). Adding GDF‐15 to a model with age, sex and LVEF improved AUC for prediction of all‐cause mortality from 0.72 (95% CI: 0.59‐0.84) to 0.79 (95% CI: 0.68‐0.91; *P* = .052; Table 3). There was no improvement of AUC when sST2 was added to the same model (AUC 0.76; 95% CI: 0.65‐0.87).

## 4 DISCUSSION

We compared the prognostic value of GDF‐15 and sST2 for prediction of fatal ventricular arrhythmias and all‐cause mortality in patients with non‐ischaemic DCM. In contrast to sST2, GDF‐15 was found to be an independent predictor of documented arrhythmic death or RCA in patients with non‐ischaemic DCM. GDF‐15 provided incremental prognostic information to LVEF in risk stratification for documented arrhythmic death or RCA. Furthermore, both GDF‐15 and sST2 were strongly associated with all‐cause mortality, and this association was independent from clinical risk factors such as LVEF and NYHA functional class. Finally, head‐to‐head comparison demonstrated that GDF‐15 was superior to sST2 in prediction of all‐cause mortality in non‐ischaemic DCM.

Prophylactic ICD implantation in patients with non‐ischaemic DCM did not improve long‐term survival in a recently published DANISH trial.^8^ However, almost twice as much patients died a sudden cardiac death in a control group as compared to the ICD group.^8^ These recent findings highlight the importance to define an improved risk stratification strategy for prophylactic ICD implantation in patients with non‐ischaemic DCM.

To best of our knowledge, this is the first study with a head‐to‐head comparison of the prognostic value of GDF‐15 and sST2 for prediction of long‐term arrhythmic and all‐cause mortality exclusively in patients with non‐ischaemic DCM. So far, only few studies have investigated GDF‐15 and sST2 in patients with non‐ischaemic DCM.^21^, ^37^ Although an association of both GDF‐15 and sST2 with sudden cardiac death was previously demonstrated, non‐ischaemic DCM represented one‐third to one‐half of these cohorts.^24^, ^25^, ^37^ Because of different aetiologies of the disease leading to different risks of AD,^5^, ^8^ it is of paramount importance to separately investigate ischaemic and non‐ischaemic heart failure patients. In addition, previous studies used sudden cardiac death as an end‐point, which is not always equivalent to fatal ventricular arrhythmia. Therefore, our primary end‐point was documented arrhythmic death or RCA (AD/RCA).

The present study demonstrates that a two‐fold increase of GDF‐15 was associated with a two‐fold higher risk of AD/RCA, whereas no association was observed for sST2. Currently, LVEF is the gold standard for risk stratification of heart failure patients, and severe LVEF reduction is an indication for prophylactic ICD implantation.^3^, ^4^ However, LVEF reflects the global systolic function of the heart, and does not necessarily correlate with pathological changes in myocardium facilitating VT. In the underlying study, GDF‐15 predicted AD/RCA independently from LVEF. It has been previously demonstrated that GDF‐15 levels correlate well with the degree of myocardial fibrosis, a substrate for the genesis of VT.^20^, ^21^ Furthermore, GDF‐15 added prognostic information on top of LVEF, which is currently the best marker for risk stratification for AD/RCA in patients with DCM. Thus, GDF‐15 might be a more specific marker for prediction of AD/RCA in non‐ischaemic DCM and provides additional information for risk stratification on top of LVEF.

sST2 is a part of IL‐33/ST2‐system, which regulates inflammation, autoimmunity, tissue repair and fibrosis,^38^, ^39^, ^40^, ^41^ and is an established prognostic biomarker in heart failure patients.^26^, ^42^ We demonstrated previously that both human macrovascular (aortic and coronary artery) and heart microvascular endothelial cells are a source for sST2 protein.^43^ IL‐33/ST2 signalling regulates cardiac remodelling after myocardial infarction and protects myocardium from hypertrophy and fibrosis.^23^, ^44^ sST2 negatively regulates IL‐33/ST2 signalling and excess sST2 promotes myocardial dysfunction and fibrosis.^22^, ^23^, ^45^ Surprisingly, several recent studies demonstrated no association between circulating sST2 levels and the degree of myocardial fibrosis.^46^, ^47^ In addition, the study by Ahmad et al demonstrated that sST2 was a better prognostic marker for heart failure than for sudden cardiac death.^37^ Thus, the prognostic value of sST2 might be its ability to predict heart failure, rather than fatal VTs in patients with non‐ischaemic DCM.^46^, ^48^

In agreement with previously published data, sST2 independently predicted all‐cause mortality in the present study. Similarly, GDF‐15 levels were also independently associated with all‐cause mortality, as was shown previously.^14^, ^17^ Uric acid was previously shown to be a powerful prognostic marker in patients with chronic heart failure, and uric acid levels were associated with GDF‐15.^14^, ^49^ In the present study, both GDF‐15 and sST2 were superior to uric acid for prediction of all‐cause mortality. In contrast to sST2, GDF‐15 predicted all‐cause mortality independently of NT‐proBNP. These results are in agreement with previous studies.^14^, ^21^ Finally, in a model including sST2 and GDF‐15, only latter remained significantly associated with all‐cause mortality in non‐ischaemic DCM patients. These results suggest that in non‐ischaemic heart failure, GDF‐15 might be superior to sST2 in predicting all‐cause mortality.

## 5 LIMITATIONS

This study had limitations: the small sample size increases the risk of type II error. Furthermore, because of the small sample size, no firm conclusion can be drawn. Regarding patients dying an AD, only LVEF was entered in a multivariable model, because of the small number of events. Furthermore, it is possible that 2 RCA might not have led to death. Therefore, findings of this study have to be confirmed in a larger sample of patients and should be labelled as pilot study results.

## 6 CONCLUSION

We demonstrated in a head‐to‐head comparison between GDF‐15 and sST2 in DCM patients the potential ability of GDF‐15 in prediction of fatal VTs and all‐cause mortality. Assessment of GDF‐15 in addition to LVEF could complement long‐term risk stratification in non‐ischaemic, DCM and thus appropriate patient selection for prophylactic ICD implantation. However, because of the small sample size, our data have to be confirmed in a larger sample of patients.

## CONFLICTS OF INTERESTS

The authors confirm that there are no conflicts of interests.
